# The impact of walking training on cognitive function in older adults: a meta-analysis

**DOI:** 10.3389/fpsyg.2026.1720121

**Published:** 2026-07-08

**Authors:** Shuang Zhao, Yuhao Zeng, Yuying Li, Shuaimin Mu, Xitang Zhao

**Affiliations:** 1 Hanjiang Normal University, Shiyan, Hubei, China; 2 Wudang Mountain International Martial Arts Academy, Wuhan Sports University, Shiyan, Hubei, China; 3 Nanjing Sport Institute, Nanjing, Jiangsu, China

**Keywords:** cognitive function, meta-analysis, older adults, RCT, walking training

## Abstract

**Objective:**

To investigate the effects of walking-based exercise on cognitive function in older adults and to determine whether different walking modalities produce improvements in specific cognitive domains.

**Methods:**

PubMed, Embase, Web of Science, and the Cochrane Library was conducted through September 9, 2025. Eligible studies were randomized controlled trials (RCTs) comparing walking interventions-such as overground, interval, or virtual-reality treadmill walking-with non-aerobic control conditions. Standardized mean differences (SMDs) and 95% confidence intervals (CIs) were calculated using fixed- or random-effects models. The *I*^2^ statistic was applied to evaluate heterogeneity, while funnel plots together with Egger’s regression analysis were used to detect potential publication bias.

**Results:**

Eight RCTs involving 772 participants (418 in the intervention and 354 in control groups) met the inclusion criteria. Walking interventions did not significantly enhance attention and executive function (SMD = 0.12, 95% CI −0.16 to 0.41; *I*^2^ = 83%), memory function (SMD = 0.04, 95% CI −0.13 to 0.21; *I*^2^ = 0%), or overall cognitive performance (SMD = 0.09, 95% CI −0.11 to 0.30; *I*^2^ = 46%). Subgroup analyses showed consistent results across different walking types. Sensitivity analyses confirmed the robustness of pooled estimates, and no publication bias was depicted (Egger’s *p* = 0.411).

**Conclusion:**

This meta-analysis found no evidence that walking training significantly improves cognitive function in older adults. These findings highlight the need for future large-scale, long-duration RCTs with standardized walking protocols, including walking speed, intensity, and domain-specific cognitive outcomes.

**Systematic review registration:**

PROSPERO; Identifier CRD420261422255.

## 1 Introduction

Cognitive decline has been identified as a concern in the context of population aging and may affect memory, attention, and executive functions that are essential for independent living. Numerous studies have identified a close link between physical and cognitive health, particularly in older adults, where mobility decline often parallels cognitive deterioration (Demnitz et al., 2018; Hoogendijk et al., 2020). In older adults, even subtle impairments in memory or executive function can substantially increase the risk of falls, loss of independence, and progression to dementia, underscoring the clinical importance of identifying modifiable factors that may preserve both mobility and cognition. Walking speed, a simple and reliable biomarker of functional aging, has been shown to predict cognitive decline and dementia risk across populations (Deshpande et al., 2009; Liu et al., 2022). Mechanistically, walking involves complex interactions among motor, sensory, and cortical systems, sharing neural substrates with higher-order cognitive processes such as planning and attention (Hall et al., 2011; Ikram et al., 2012). Because walking is a daily activity that integrates motor control, attentional allocation, and environmental monitoring, impairments in walking performance may reflect early dysfunction in cognitive networks critical for independent aging. Thus, interventions that target walking capacity may simultaneously enhance cognitive function and overall brain health.

Accumulating evidence supports the beneficial role of physical activity in maintaining or improving cognitive performance in late life. Aerobic exercise, in particular, enhances neuroplasticity through increased cerebral blood flow, neurogenesis, and upregulation of brain-derived neurotrophic factors (Kuo et al., 2022; Fettrow et al., 2021). Among various exercise modalities, walking stands out for its accessibility, safety, and adaptability to diverse older populations (Karmakar et al., 2023; Kim et al., 2021). Unlike more complex or equipment-dependent exercise programs, walking can be easily implemented at the community level and sustained over long periods, making it particularly relevant for older adults with functional limitations. Clinical trials have demonstrated that walking interventions can improve attention, processing speed, and executive functioning, though results remain inconsistent depending on exercise intensity, supervision, and participant cognitive status (Jamrasi et al., 2024; Grede et al., 2024). These discrepancies highlight the need for a comprehensive synthesis of available evidence to clarify the cognitive benefits of walking in older adults.

Recent meta-analytic and longitudinal studies further indicate that walking performance is closely associated with multiple cognitive domains, including executive function, memory, processing speed, and global cognition. For example, concurrent decline in walking speed and memory strongly predicts dementia risk (Collyer et al., 2022), while slower walking speed has been prospectively linked to reduced executive function and global cognitive performance (Mielke et al., 2013; Taniguchi et al., 2012). Importantly, these associations are domain-specific, suggesting that different cognitive systems may be differentially sensitive to changes in walking performance. These domain-specific associations suggest that walking-based interventions may differentially influence cognitive processes. Executive function may benefit through increased prefrontal activation during motor-cognitive coordination tasks, memory may improve through aerobic exercise-induced hippocampal plasticity, and processing speed may improve via enhanced cerebral perfusion.

To ensure conceptual clarity and methodological consistency across the manuscript, this study focuses on the following cognitive domains commonly assessed in exercise–cognition research: (1) attention and executive function; (2) memory; (3) processing and reaction speed; (4) dual-task performance, reflecting simultaneous motor–cognitive demands; (5) drawing and visuospatial–executive tasks; (6) substitution and symbol tasks reflecting processing efficiency; and (7) global cognitive screening. These domains align with prior neuropsychological frameworks and with empirical evidence linking gait to domain-specific cognitive decline (Soumare et al., 2009; Wang et al., 2022).

Recent meta-analytic and interventional studies suggest that walking training, including overground, treadmill, and virtual-reality-based protocols, may contribute to cognitive resilience, particularly among those with mild cognitive impairment (Kang et al., 2025; Li et al., 2024). However, findings are heterogeneous across cognitive domains and study designs. Some trials report significant improvements in executive or dual-task performance, whereas others observe minimal cognitive gains (Kemoun et al., 2010; Hackney et al., 2015). Furthermore, few systematic reviews have quantitatively assessed the extent of cognitive enhancement attributable solely to walking, independent of multimodal training. Therefore, this meta-analysis aims to synthesize randomized controlled trials (RCTs) to determine the overall impact of walking training on cognitive function among older adults, with subgroup analyses exploring differences across intervention types and cognitive domains.

## 2 Methods

### 2.1 Literature search strategy

A thorough search of the PubMed, Embase, Web of Science, and Cochrane Library databases was performed to search eligible studies published before September 9, 2025. The search combined Medical Subject Headings (MeSH) and free-text terms related to walking, cognitive function, and older populations. The complete PubMed search strategy was as follows:

(((((Walking [MeSH Terms]) OR (Walking training [Title/Abstract])) OR (Walking [Title/Abstract])) OR (Ambulation [Title/Abstract])) AND ((((((((((Cognition [MeSH Terms]) OR (Cognition [Title/Abstract])) OR (Cognitions [Title/Abstract])) OR (Cognitive Function [Title/Abstract])) OR (Cognitive Functions [Title/Abstract])) OR (Function, Cognitive [Title/Abstract])) OR (Functions, Cognitive [Title/Abstract])) OR (Insight [Title/Abstract])) OR (Insights [Title/Abstract])) OR (Memory [Title/Abstract]))) AND (((Aged [MeSH Terms]) OR (Aged [Title/Abstract])) OR (Elderly [Title/Abstract]))

Equivalent search strategies were adapted for the other databases (Supplementary Table S1). In addition, the reference sections of the included articles and related reviews were manually examined to find other studies that met the eligibility criteria. This systematic review and meta-analysis was registered in PROSPERO with the registration number CRD420261422255. The study was conducted and reported in accordance with the Preferred Reporting Items for Systematic Reviews and Meta-Analyses (PRISMA) guidelines.

### 2.2 Inclusion and exclusion criteria

Studies were considered eligible for inclusion if they satisfied the following conditions:

**Population:** Participants classified as older adults (generally aged ≥65 years), including healthy older adults and those with mild cognitive impairment (MCI).

**Intervention:** Walking-based interventions in which walking was included as a core component of the intervention, including overground walking, treadmill walking, interval walking training, and walking exercise. Interventions were eligible whether delivered individually or in supervised group-based formats, and whether walking was implemented alone or combined with other components (e.g., resistance exercise, cognitive tasks, or environmental enrichment).

Walking-based interventions incorporating additional motor–cognitive or environmental features, such as virtual reality treadmill training (VRTT) or dual-task walking paradigms, were also eligible.

**Comparator:** Usual care, no intervention, waiting-list control, or other control conditions that did not include walking training (e.g., health education, stretching, yoga, or sedentary activities).

**Outcomes:** Quantitative measures of cognitive function assessed using validated instruments.

**Study design:** RCTs.

No language restrictions were applied.

**Exclusion criteria:**

Studies were excluded if (1) walking training was not included in the intervention group, (2) the control group included walking training, (3) they were conference abstracts, reviews, case reports, protocols, or animal studies, or (4) sufficient data for effect size calculation were unavailable.

### 2.3 Data extraction and quality assessment

Two investigators independently screened and extracted data using a predefined standardized form. The extracted information included: study characteristics (first author, publication year, and country), study type, sample size, mean age, gender distribution, intervention details (mode, frequency, intensity, and duration), control condition, follow-up period, gait speed (if reported), and cognitive outcomes. Disagreements between reviewers were settled through discussion or, when necessary, by consulting a third evaluator. The quality of each included randomized trial was evaluated using the Cochrane Risk of Bias version 2.0 (RoB 2) tool (Martimbianco et al., 2023). The RoB 2 tool assesses five key areas of potential bias: (1) bias related to the randomization process, (2) bias resulting from deviations from the intended interventions, (3) bias caused by incomplete outcome data, (4) bias concerning the measurement of outcomes, and (5) bias associated with the selection of reported results. Each domain was categorized as low risk, raising some concerns, or high risk, and an overall risk judgment was made for every included study. Cognitive outcomes were grouped for meta-analysis based on similarities in task demands, test structure, and outcome measurement characteristics, rather than on strict or mutually exclusive cognitive-domain or neuroanatomical classifications. We acknowledge that many cognitive tasks engage overlapping cognitive processes and distributed neural networks, particularly for executive and attentional components. Therefore, the purpose of this grouping strategy was to facilitate statistically meaningful synthesis of comparable outcome measures, rather than to define discrete cognitive domains.

### 2.4 Statistical analysis

All data analyses were carried out with Stata software (version 18.0; StataCorp, College Station, TX, USA) and Review Manager (RevMan, version 5.4). For continuous variables, standardized mean differences (SMDs) along with 95% confidence intervals (CIs) were computed to compare participants in the walking intervention and control groups. When only change-from-baseline information was provided, appropriate conversion equations were used to standardize the data across studies.

The random-effects model was applied to address possible heterogeneity among studies. Statistical variability was evaluated through Cochran’s *Q* test and quantified using the *I*^2^ statistic, with values exceeding 50% considered indicative of notable heterogeneity. To test the robustness of the combined estimates, sensitivity analyses were performed by omitting studies at high risk of bias or by reanalyzing the results using alternative modeling strategies (fixed-effect versus random-effect models).

All cognitive outcomes extracted from the included trials were grouped into seven domains based on established neuropsychological frameworks and widely accepted distinctions in exercise–cognition research. Attention and executive function were categorized together because these processes rely on overlapping prefrontal–cortical networks responsible for inhibitory control, cognitive flexibility, and attentional shifting, as described in the unity-and-diversity model of executive functions (Miyake et al., 2000; Alvarez and Emory, 2006). These outcomes were assessed through Stroop-related tasks and composite executive-function measures. Memory outcomes—including episodic and working memory—were classified as a separate domain, reflecting their reliance on hippocampal and medial temporal lobe structures, consistent with established models of memory and working-memory function (Squire and Zola, 1996; Baddeley, 2012), and were measured using delayed-recall and sequencing tasks. Processing and reaction speed were grouped together as indicators of cognitive efficiency and psychomotor responsiveness, following Salthouse’s processing-speed theory (Salthouse, 1996), and were assessed using EEG peak frequency, reaction-time tasks, and coding tests. Dual-task performance was treated as an independent domain because it reflects motor–cognitive interference and attentional resource allocation during simultaneous task demands, consistent with dual-task interference literature (Pashler, 1994; Woollacott and Shumway-Cook, 2002), and was quantified using dual-task effects on reaction time (DTERT), dual-task effects on accuracy (DTEACC), and dual-task effects on cognitive task performance (DTECT) measures. Drawing and visuospatial–executive tasks, represented by the Clock Drawing Test, were classified separately due to their integration of planning, visuoconstruction, and visuospatial processing, following established neuropsychological interpretations of the test (Shulman, 2000). Substitution and symbol tasks—including the Digit-Symbol Substitution Test (DSST) and the Yamaguchi Kanji–Symbol Substitution Test (YKSST)—were grouped as measures of processing speed and associative learning, consistent with the test’s known sensitivity to age-related cognitive decline (Joy et al., 2003). Finally, global cognitive screening tools (MoCA, MMSE, Color–Word Stroop Test, and DCCS) were categorized together because they assess multiple cognitive systems simultaneously and provide a general index of overall cognitive status (Folstein et al., 1975; Nasreddine et al., 2005). This classification aligns with well-established neuropsychological theory and supports consistent synthesis of heterogeneous cognitive outcomes across trials.

### 2.5 Publication bias

Possible publication bias was assessed both visually, through funnel plots, and statistically, by applying Egger’s regression analysis in Stata software. Egger’s test was performed only when ten or more studies were included to evaluate potential small-study effects. If the funnel plot displayed noticeable asymmetry implying such effects, the trim-and-fill procedure was employed to generate an adjusted pooled effect estimate.

## 3 Results

### 3.1 Literature retrieval, study characteristics, and quality evaluation

**Figure 1:**
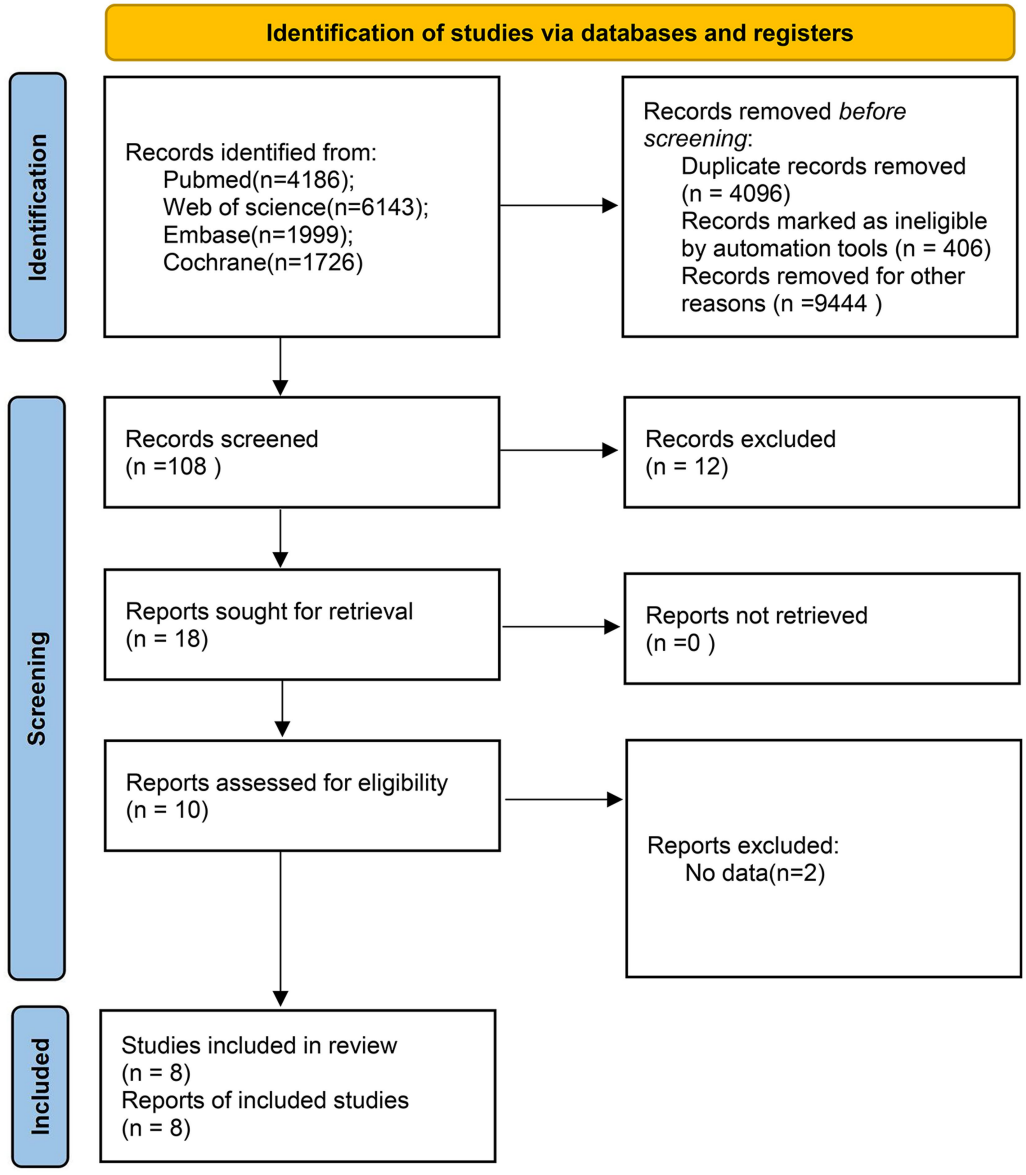
PRISMA flow diagram of literature search and study selection.

A total of 14,054 records were retrieved from database searches, comprising 4,186 from PubMed, 6,143 from Web of Science, 1,999 from Embase, and 1,726 from the Cochrane Library. After removing 4,096 duplicates, 9,958 studies were screened by title and abstract. Based on study type, 406 records-including reviews, conference abstracts, study protocols, case reports, and animal experiments-were excluded. Following full-text assessment, additional articles were excluded for being unrelated to the research topic or lacking extractable outcome data. Finally, eight RCTs (Karmakar et al., 2023; Jamrasi et al., 2024; Grede et al., 2024; Maki et al., 2012; Okamoto et al., 2019; Oken et al., 2006; Zheng et al., 2024; Zukowski et al., 2022) met the eligibility criteria and were included in the meta-analysis (Figure 1). These trials involved 772 participants in total, with 418 assigned to walking intervention groups and 354 to control groups (Table 1). Walking dose parameters are summarized in Supplementary Table S2.

**Table 1:** Summary of the basic characteristics of the included studies.

Study	Country	Study type	Sample size	Age	Gender (F/M)	Intervention	Control	Follow-up time	Gait speed	Outcome(s)
Oken et al. (2006)	USA	Randomized controlled trial	Exercise: 47	65–85	Exercise: 37/10	Walking exercise	Yoga; Wait list	6 months	NA	Stroop interference (sec); EEG auditory MPF (Hz); word list delayed recall; letter-number sequencing; covert orienting (invalid–valid); divided attention threshold (ms); % errors above threshold; set shifting: highest shift; simple reaction time (ms); choice reaction time (ms)
			Yoga: 44	Yoga: 31/13						
			Wait list: 44	Wait list: 33/11						
Okamoto et al. (2019)	Japan	Randomized controlled trial	Interval walking training: 22	Interval walking training: 72 ± 1	Interval walking training: 11/11	Interval walking training	Normal walking	20 weeks	NA	TMT-A; TMT-B
			Normal walking training: 24	Normal walking training: 73 ± 1	Normal walking training:14/10					
Maki et al. (2012)	Japan	Randomized controlled trial	Walking training: 75	Walking training: 71.9 ± 4.1	Walking training: 52/23	Walking training	Educational lecture	7 days	NA	Category cued delayed recall test; Dual-Task Test; Trail-Making Test (TMT, Part A & B); Clock Drawing Test; Digit-Symbol Substitution Test (DSST, WAIS-III); Yamaguchi Kanji-Symbol Substitution Test (YKSST)
			Educational lecture: 75	Educational lecture: 72.0 ± 3.9	Educational lecture: 54/21					
Zukowski et al. (2022)	USA	Randomized controlled trial	Virtual reality treadmill training (VRTT): 30	Virtual reality treadmill training (VRTT): 71.2 ± 6.5	Virtual reality treadmill training (VRTT): 22/8	Virtual reality treadmill training (VRTT)	Conventional treadmill training (CTT)	NA	10 Meter Walk Test (m/s)	Montreal Cognitive Assessment (MoCA); WAIS-IV Coding; Comprehensive Trail Making Test; Stroop Color-Word Interference Test; Cognitive response reaction time (ms); Cognitive response accuracy (%); Cognitive throughput (correct responses per minute); DTERT (%); DTEACC (%); DTECT (%)
			Conventional treadmill training (CTT): 30	Conventional treadmill training (CTT): 72.0 ± 7.7	Conventional treadmill training (CTT): 21/9				Virtual reality treadmill training (VRTT): 1.4 (1.2–1.5)	
									Conventional treadmill training (CTT): 1.3 (1.2–1.4)	
Karmakar et al. (2023)	Saudi Arabia	Randomized controlled trial	Supervised group-based intervention (SGBI): 42	Supervised group-based intervention (SGBI): 62.37 ± 4.89	Supervised group-based intervention (SGBI): 17/25	Supervised group-based intervention (SGBI)	Non-supervised	15 weeks	NA	Mini-Mental State Examination (MMSE)
			Non-supervised	Non-supervised	Non-supervised		Individual-based intervention (NSIBI)			
			Individual-based intervention (NSIBI): 36	Individual-based intervention (NSIBI): 61.66 ± 3.49	Individual-based intervention (NSIBI): 15/21					
Grede et al. (2024)	Germany	Randomized controlled trial	Walking training: 114	Walking training:85.00 (79.0–90.0)	NA	Walking training	Educational lecture	12 months	NA	Mini-Mental State Examination (MMSE); Clock Drawing Test (CDT)
			Educational lecture: 110	Educational lecture: 84.00(80.0,90.0)						
Jamrasi et al. (2024)	Korea	Randomized controlled trial	Resistance + Walking: 25	Resistance + Walking: 73.0 ± 4.6	Resistance + Walking: 20/5	Resistance + Walking; Walking	Control	12 weeks	NA	Mini-Mental State Examination (MMSE); Color-Word Stroop Test (CWST, Korean version)
			Walking:27	Walking:73.9 ± 4.2	Walking: 15/12					
			Control: 27	Control: 74.3 ± 5.9	Control: 19/8					
Zheng et al. (2024)	USA	Randomized crossover study	26	Walking: 65.4 ± 11.7	17/9	Walking	Sitting	NA	Average speed of 1.12 m/s	Flanker Inhibitory Control and Attention Test; Dimensional Change Card Sort Test (DCCS); Pattern Comparison Processing Speed Test (PCPS)
				Sitting: 72.3 ± 9.4						

**Figure 2:**
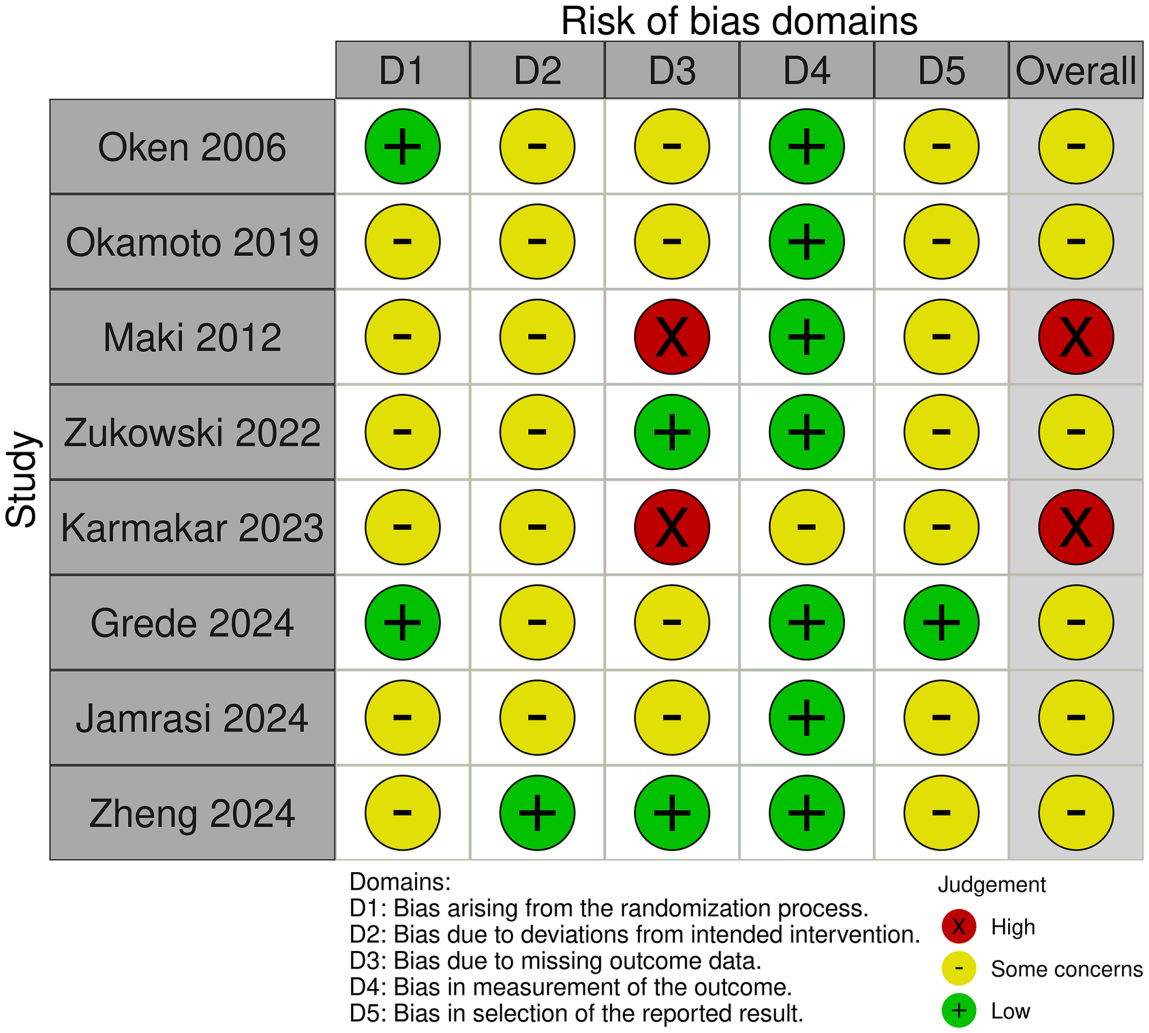
Quality assessment of included studies using the Cochrane Risk of Bias tool.

The methodological quality of the included studies was assessed using the Cochrane Risk of Bias version 2.0 (RoB 2) tool, and the domain-specific evaluations are illustrated in Figure 2. Overall, most trials demonstrated some concerns of bias, while two studies (Karmakar et al., 2023; Maki et al., 2012) were rated as high risk of bias. For the randomization process, two studies (Grede et al., 2024; Oken et al., 2006) were judged as low risk, whereas the others presented some concerns due to incomplete reporting of allocation procedures. All studies were rated as some concerns for deviations from intended interventions, as participant blinding was impractical for behavioral training. Regarding missing outcome data, Karmakar et al. (2023) and Maki et al. (2012) were classified as high risk owing to attrition or incomplete follow-up, while other trials adequately reported outcomes. The measurement of the outcome was judged as low risk in most studies (Grede et al., 2024; Okamoto et al., 2019; Oken et al., 2006; Zheng et al., 2024; Zukowski et al., 2022), since validated cognitive tests were used and outcome assessors were frequently blinded. All studies showed some concerns in selection of the reported results because prespecified outcomes were not consistently registered or fully reported. In summary, seven trials were rated as having some concerns overall, and two trials (Karmakar et al., 2023; Maki et al., 2012) were classified as high risk of bias. These findings indicate that the methodological quality of the included studies was generally acceptable, although bias related to intervention deviations and selective reporting may have affected the robustness of some estimates, and the pooled evidence should be interpreted with appropriate caution.

### 3.2 Meta-analysis results

#### 3.2.1 Meta-analysis of attention and executive-related task

Five RCTs (Maki et al., 2012; Okamoto et al., 2019; Oken et al., 2006; Zheng et al., 2024; Zukowski et al., 2022) evaluated the effects of walking-based interventions on attention and executive function in older adults. In this review, the term walking-based interventions is used as an umbrella concept encompassing programs described in the original studies as walking exercise, interval walking training, walking training, and virtual-reality treadmill training (VRTT). Although the terminology varied across studies, these interventions shared a common core component of structured or prescribed walking, differing mainly in intensity modulation, training context, or the use of technological augmentation. Control conditions included yoga, wait-list control, normal walking, educational lectures, conventional treadmill training (CTT), or sitting.

**Figure 3:**
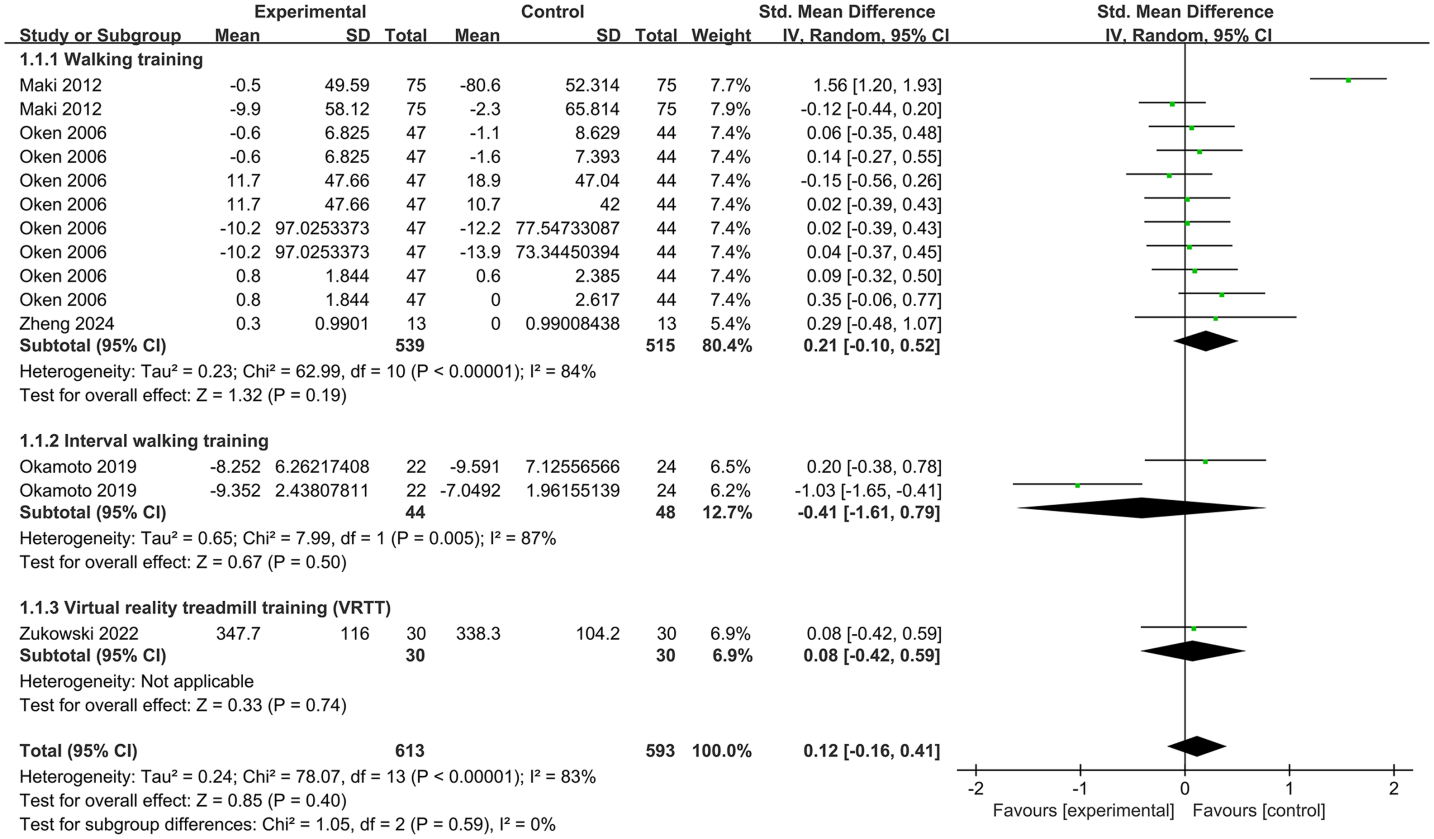
Forest plot of the effect of walking-based interventions on attention and executive function in older adults.

As shown in Figure 3, the pooled analysis using a random-effects model indicated that walking-based exercise did not significantly improve attention or executive function compared with control interventions (SMD = 0.12; 95% CI −0.16 to 0.41; *p* = 0.40). Substantial heterogeneity was observed (*I*^2^ = 83%, *p* < 0.00001).

Subgroup analysis showed that walking training had an SMD of 0.21 (95% CI −0.10 to 0.52; *p* = 0.19; *I*^2^ = 84%), interval walking training had an SMD of −0.41 (95% CI −1.61 to 0.79; *p* = 0.50; *I*^2^ = 87%), and virtual-reality treadmill training (VRTT) had an SMD of 0.08 (95% CI −0.42 to 0.59; *p* = 0.74). No significant subgroup difference was observed (*p* = 0.59; *I*^2^ = 0%). No significant subgroup difference was observed (*p* = 0.59; *I*^2^ = 0%).

#### 3.2.2 Meta-analysis of memory-related task

Two RCTs (Maki et al., 2012; Oken et al., 2006) investigated the effect of walking-based exercise on memory function in older adults. The intervention groups performed walking exercise or structured walking training, while the control groups engaged in yoga, were placed on a wait-list, or attended educational lectures. Memory performance was evaluated using standardized cognitive tests, including the Word-List Delayed Recall, Letter-Number Sequencing, and Delayed Recall tasks, which assess episodic and working-memory ability.

**Figure 4:**
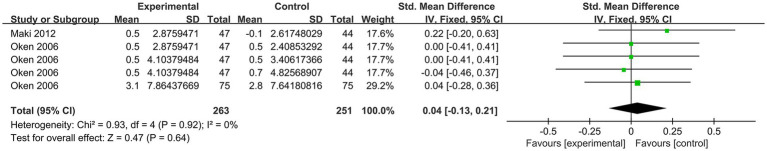
Forest plot of the effect of walking-based interventions on memory function.

As presented in Figure 4, the pooled analysis using a fixed-effects model showed no significant improvement in memory function following walking-based training compared with control interventions (SMD = 0.04; 95% CI: −0.13 to 0.21; *p* = 0.64). Heterogeneity was negligible (*I*^2^ = 0%, *p* = 0.92), indicating consistent results across the included studies. Although both trials reported slight increases in recall and sequencing scores after walking exercise, these effects were not statistically significant.

#### 3.2.3 Meta-analysis of processing speed and reaction time-related outcomes

Three RCTs (Oken et al., 2006; Zheng et al., 2024; Zukowski et al., 2022) evaluated the effects of walking-based interventions on processing speed and reaction time in older participants. The intervention programs included walking exercise, structured walking training, and virtual-reality treadmill training (VRTT), while the control groups engaged in yoga, wait-list conditions, conventional treadmill training (CTT), or sitting.

Processing and reaction speed were assessed using several validated neuropsychological and neurophysiological measures, including EEG Auditory Mean Peak Frequency (MPF, Hz), Simple Reaction Time (ms), Choice Reaction Time (ms), WAIS-IV Coding, and the Pattern Comparison Processing Speed Test (PCPS).

**Figure 5:**
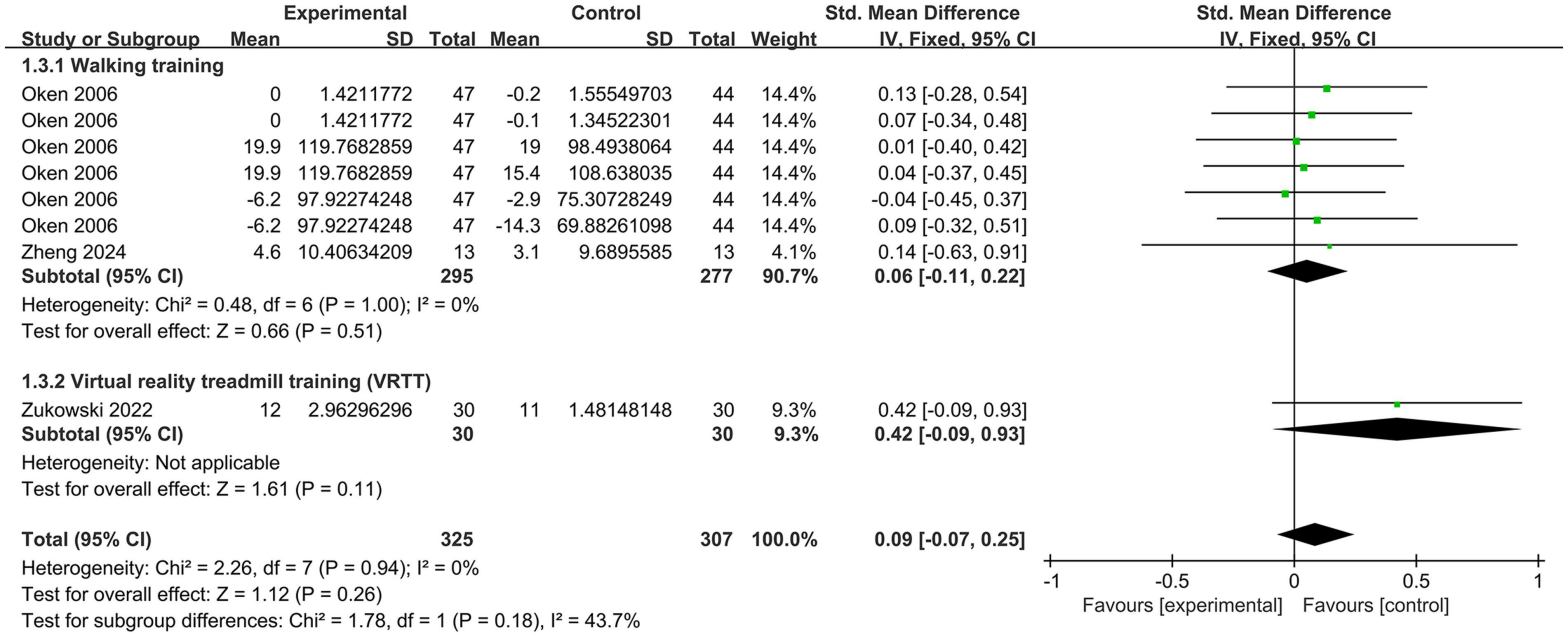
Forest plot of the effect of walking-based interventions on processing speed and reaction time.

As shown in Figure 5, the overall pooled analysis using a fixed-effects model indicated no statistically significant improvement in processing or reaction speed following walking-based interventions compared with control conditions (SMD = 0.09; 95% CI: −0.07 to 0.25; *p* = 0.26). Heterogeneity was negligible (*I*^2^ = 0%, *p* = 0.94), suggesting consistency among studies.

Subgroup analysis showed that walking training had an SMD of 0.06 (95% CI: −0.11 to 0.22; *p* = 0.51; *I*^2^ = 0%), while virtual-reality treadmill training (VRTT) had an SMD of 0.42 (95% CI: −0.09 to 0.93; *p* = 0.11). No significant subgroup difference was observed (*p* = 0.18; *I*^2^ = 43.7%).

#### 3.2.4 Meta-analysis of dual-task performance

Two RCTs (Maki et al., 2012; Zukowski et al., 2022) evaluated the effects of walking-based exercise on dual-task performance in older adults. The intervention programs included walking training and virtual-reality treadmill training (VRTT), while the control groups received educational lectures or conventional treadmill training (CTT).

Dual-task performance was assessed using the Dual-Task Test, DTERT (%), DTEACC (%), and DTECT (%).

**Figure 6:**
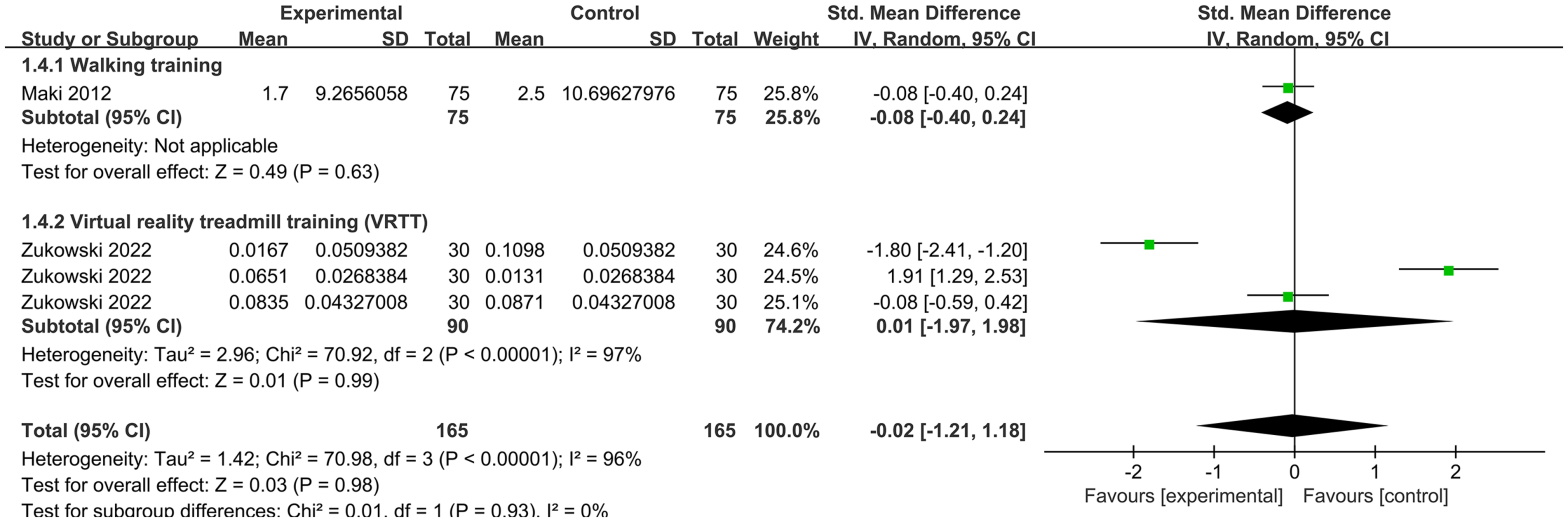
Forest plot of the effect of walking-based interventions on dual-task performance.

As shown in Figure 6, the pooled analysis using a random-effects model demonstrated no significant difference in dual-task performance between the intervention and control groups (SMD = −0.02; 95% CI: −1.21 to 1.18; *p* = 0.98). Substantial heterogeneity was observed (*I*^2^ = 96%, *p* < 0.00001).

Subgroup analysis showed that walking training had an SMD of −0.08 (95% CI: −0.40 to 0.24; *p* = 0.63), while virtual-reality treadmill training (VRTT) had an SMD of 0.01 (95% CI: −1.97 to 1.98; *p* = 0.99; *I*^2^ = 97%). No significant subgroup difference was found (*p* = 0.93; *I*^2^ = 0%).

#### 3.2.5 Meta-analysis of visuoconstructive executive task

Two RCTs (Grede et al., 2024; Maki et al., 2012) evaluated the effect of walking-based interventions on drawing and executive-function tasks in older participants. The intervention groups performed walking training, while the control groups received educational lectures.

Executive and visuospatial function in these studies was assessed using the Clock Drawing Test, which measures planning, attention, and visuoconstructional ability within executive-cognitive domains.

**Figure 7:**
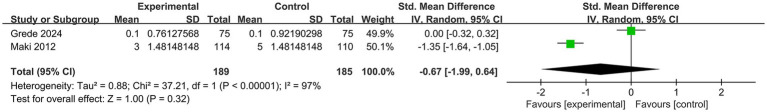
Forest plot of the effect of walking-based interventions on drawing and executive tasks.

As shown in Figure 7, the pooled analysis using a random-effects model indicated no significant difference between the walking-training and control groups (SMD = −0.67; 95% CI: −1.99 to 0.64; *p* = 0.32). Substantial heterogeneity was observed (*I*^2^ = 97%, *p* < 0.00001), suggesting variability between studies in intervention duration, participant characteristics, and cognitive assessment conditions.

Although one trial (Maki et al., 2012) reported partial improvement in executive-drawing performance, the overall pooled result showed that walking-based training did not significantly enhance performance on drawing or executive-function tasks compared with non-exercise control conditions.

#### 3.2.6 Meta-analysis of symbol substitution tasks

One randomized controlled trial (Maki et al., 2012) examined the effect of walking-based exercise on substitution and symbol-processing tasks in older adults. The intervention group participated in walking training, while the control group received educational lectures.

Cognitive processing speed and symbol-number association ability were assessed using the Digit-Symbol Substitution Test (DSST, WAIS-III) and the Yamaguchi Kanji-Symbol Substitution Test (YKSST), a version adapted for older adults.

**Figure 8:**
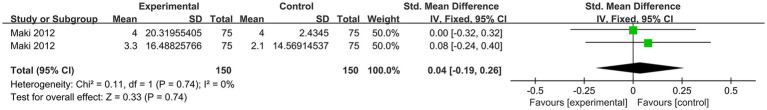
Forest plot of the effect of walking-based interventions on substitution and symbol tasks.

As shown in Figure 8, the pooled analysis using a fixed-effects model revealed no significant difference in substitution and symbol-task performance between the walking-training and control groups (SMD = 0.04; 95% CI: −0.19 to 0.26; *p* = 0.74). Heterogeneity was negligible (I^2^ = 0%, p = 0.74), indicating consistent results between the two included measures.

These findings suggest that walking-based training did not produce measurable improvement in substitution or symbol-processing performance compared with educational lecture controls. Both intervention and control groups showed comparable stability in processing speed and symbol-association abilities.

#### 3.2.7 Meta-analysis of global cognitive screening measures

Three RCTs (Karmakar et al., 2023; Grede et al., 2024; Zukowski et al., 2022) evaluated the impact of walking-based interventions on global cognitive function in older adults. The intervention programs included virtual reality treadmill training (VRTT), supervised group-based intervention (SGBI), and walking training, while the control groups received conventional treadmill training (CTT), individual-based intervention (NSIBI), or educational lectures.

Global cognitive performance was assessed using standardized instruments, including the Montreal Cognitive Assessment (MoCA), Mini-Mental State Examination (MMSE), Color-Word Stroop Test (CWST, Korean version), and Dimensional Change Card Sort Test (DCCS).

**Figure 9:**
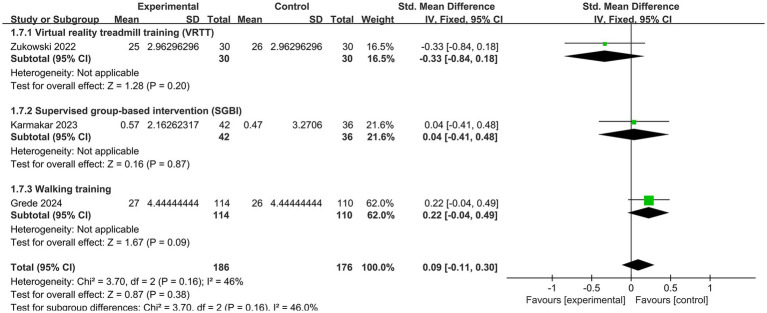
Forest plot of the effect of walking-based interventions on global cognitive screening outcomes.

As shown in Figure 9, the pooled analysis using a fixed-effects model demonstrated no significant difference in global cognitive outcomes between the intervention and control groups (SMD = 0.09; 95% CI: −0.11 to 0.30; *p* = 0.38). Heterogeneity was moderate (*I*^2^ = 46%, *p* = 0.16), indicating acceptable consistency among studies.

Subgroup analysis showed that virtual reality treadmill training (VRTT) had an SMD of −0.33 (95% CI: −0.84 to 0.18; *p* = 0.20), supervised group-based intervention (SGBI) had an SMD of 0.04 (95% CI: −0.41 to 0.48; *p* = 0.87), and walking training had an SMD of 0.22 (95% CI: −0.04 to 0.49; *p* = 0.09). No significant subgroup difference was observed (*p* = 0.16; *I*^2^ = 46.0%).

#### 3.2.8 Meta-analysis results of high-risk studies have been removed

For attention and executive function, excluding high-risk studies did not change the non-significant result (SMD = 0.04; 95% CI: −0.09 to 0.17; *p* = 0.58), but reduced heterogeneity from high to non-significant levels (*I*^2^: 83–29%). (Table 2).

**Table 2:** The meta-analysis results of high-risk studies have been removed.

Outcome	Number of included trails	Intervention (control vs treatment groups)	Sample (control vs treatment groups)	SMD [95%CI]	*p* value	*I* ^2^
Attention & executive function	12 (Okamoto et al., 2019; Oken et al., 2006; Zheng et al., 2024; Zukowski et al., 2022)	Yoga, wait list, normal walking training, CTT, sitting vs. exercise, interval walking, VRTT, Walking	443 vs. 463	0.04 [−0.09, 0.17]	0.58	29%
Memory function	4 (Oken et al., 2006)	Yoga, wait list vs. exercise	176 vs. 188	0.04 [−0.16, 0.25]	0.68	0%
Dual-task performance	3 (Zukowski et al., 2022)	Education lecture, CTT vs. VRTT	90 vs. 90	0.01 [−1.97, 1.98]	0.99	97%
Global cognitive screening	2 (Grede et al., 2024; Zukowski et al., 2022)	CTT, educational lecture vs. VRTT	140 vs. 144	−0.01 [−0.55, 0.53]	0.97	72%

For memory function, excluding high-risk studies did not change the non-significant result (SMD = 0.04; 95% CI: −0.16 to 0.25; *p* = 0.68), and heterogeneity remained absent (*I*^2^ = 0%). Compared with the original analysis (SMD = 0.04; 95% CI: −0.13 to 0.21; *p* = 0.64; I^2^ = 0%), the pooled effect size and overall conclusion were essentially unchanged (Table 2).

For dual-task performance, after excluding high-risk studies, the pooled result remained non-significant (SMD = 0.01; 95% CI: −1.97 to 1.98; *p* = 0.99), with high heterogeneity still observed (*I*^2^ = 97%). Compared with the original analysis (SMD = −0.02; 95% CI: −1.21 to 1.18; *p* = 0.98; *I*^2^ = 96%), the effect estimate and conclusion were essentially unchanged (Table 2).

For global cognitive screening, after excluding high-risk studies, the pooled result remained non-significant (SMD = −0.01; 95% CI: −0.55 to 0.53; *p* = 0.97), but heterogeneity increased to a high level (*I*^2^ = 72%). Compared with the original analysis (SMD = 0.09; 95% CI: −0.11 to 0.30; *p* = 0.38; *I*^2^ = 46%), the overall conclusion remained unchanged, indicating no significant effect of walking-based interventions on Global Cognitive Screening (Table 2).

### 3.3 Publication bias and sensitivity analysis

The funnel plot appeared generally symmetrical, indicating no significant publication bias among the included studies (Supplementary Figure S1). Similarly, Egger’s test for Attention and Executive Function showed no evidence of publication bias (*p* = 0.411). Sensitivity analysis revealed that omitting any single study did not materially alter the overall effect size, confirming that the pooled results were robust and stable across all included studies (Supplementary Figure S2).

## 4 Discussion

This meta-analysis synthesized evidence from nine RCTs involving 772 older participants to evaluate the effects of walking-based interventions on multiple domains of cognitive function. The pooled results demonstrated that walking training did not produce statistically significant improvements in attention, executive function, memory, processing speed, dual-task performance, drawing and executive tasks, substitution and symbol tasks, or global cognitive screening when compared with non-aerobic controls such as yoga, education, or waiting-list conditions. Importantly, this absence of statistically significant effects represents the primary finding of the present study and warrants careful interpretation rather than being viewed as evidence of inefficacy. Subgroup analyses across intervention types-walking training, interval walking, and VRTT—revealed no significant differences, although certain trials showed domain-specific tendencies toward improved executive or global cognition. Sensitivity analyses confirmed the robustness of findings, with no materially deviating studies identified, and no publication bias was detected. Nevertheless, because some included studies raised concerns regarding deviations from intended interventions and selective reporting, these potential biases may still have affected the certainty of some estimates and should be considered when interpreting the conclusions. Collectively, these results suggest that walking alone, while physiologically beneficial, may exert limited or subtle effects on higher-order cognitive performance in older adults, particularly when cognitive outcomes are measured using diverse instruments and intervention heterogeneity remains high. Taken together, these null findings suggest that walking alone may exert limited or highly variable effects on higher-order cognitive performance in older adults, particularly when intervention characteristics and outcome assessments are heterogeneous.

The results of this meta-analysis align with prior systematic reviews and interventional studies showing mixed or modest cognitive effects of walking-based exercise among older adults. Several large-scale trials have suggested that aerobic exercise can improve specific cognitive domains, particularly executive and attentional functions, by enhancing cerebral perfusion and neuroplasticity (Voss et al., 2010; Erickson et al., 2010). However, walking interventions—when delivered as a single, low-complexity modality—often yield smaller effect sizes than structured, multi-component aerobic or cognitively enriched exercise programs (Kelly and Shumway-Cook, 2014). This distinction is critical for interpreting the present findings, as walking alone may not provide sufficient cognitive or physiological challenge to elicit measurable improvements across multiple cognitive domains. Consistent with our findings, Jamrasi et al. (2024) reported that moderate-intensity walking training improved physical fitness but failed to significantly enhance executive function in healthy older adults. Similarly, Karmakar et al. (2023) and Grede et al. (2024) observed no significant differences in global cognitive outcomes between walking-based and conventional control interventions. These observations collectively suggest that walking may be sufficient to maintain general health and mobility, but may not provide an adequate cognitive stimulus unless combined with sufficient intensity or cognitive challenge.

Neurobiological evidence provides a plausible explanation for the modest cognitive gains observed. Walking is a rhythmic, automatic motor activity that places relatively low cognitive demand on cortical control systems compared with more complex or cognitively engaging exercise modalities (Kuo et al., 2022; Fettrow et al., 2021). Exercise modalities incorporating cognitive engagement-such as dance, exergaming, or dual-task treadmill walking-have been shown to stimulate prefrontal activation and improve attention and w\orking memory more effectively (Eggenberger et al., 2015). In the present analysis, walking interventions varied widely in duration (6–24 weeks), intensity, supervision, and task complexity, and most lacked explicit cognitive components or progressive overload, which may have limited their capacity to induce measurable neuroplastic adaptations. Furthermore, studies employing neuroimaging have demonstrated that aerobic intensity, rather than exercise type alone, mediates hippocampal volume increases and white-matter integrity (Maleki et al., 2022; Zhang et al., 2024). Thus, the null findings observed in this meta-analysis may reflect insufficient intervention intensity or cognitive load rather than a true absence of a walking–cognition relationship. Accordingly, interventions limited to low or moderate-intensity walking may not reach the physiological threshold required for detectable cognitive change.

Although pooled results indicated no significant overall effect, some domain-specific trends merit cautious interpretation. For attention and executive function, walking training yielded a small but non-significant positive trend (SMD = 0.21), which resonates with prior work suggesting that these domains are more responsive to aerobic interventions due to shared neural pathways between motor coordination and executive control (Liu et al., 2022; Hall et al., 2011). Conversely, interval walking training showed inconsistent effects and substantial heterogeneity (*I*^2^ = 87%), possibly reflecting variation in intensity protocols and adherence rates. VRTT showed slight improvement trends in global cognition, which may relate to enhanced sensorimotor integration and attentional engagement provided by immersive environments (Kang et al., 2025; Zukowski et al., 2022). Importantly, these subgroup analyses were exploratory in nature and were not adjusted for multiple comparisons; therefore, any apparent domain- or intervention-specific trends should be interpreted with caution. However, none of these subgroup differences reached statistical significance, and given the small sample sizes, these findings should be interpreted as exploratory rather than confirmatory.

Several methodological factors further contributed to the null findings. Many included trials recruited fewer than 60 participants, limiting statistical power to detect small-to-moderate cognitive effects. Substantial heterogeneity in intervention dose (frequency, duration, and intensity) and cognitive assessment tools further diluted potential effects. Widely used screening tools such as the MoCA and MMSE may lack sensitivity to detect subtle changes in executive or processing functions (Fiorenzato et al., 2016; Ismail et al., 2019), whereas studies using more specific neuropsychological tests (e.g., Stroop or TMT-B) appeared more responsive to intervention-related change. This suggests that the observed null findings may partly reflect limitations of outcome measurement rather than an absence of meaningful cognitive change. This underscores the importance of outcome selection in exercise–cognition research and suggests that non-significant findings may partly reflect measurement insensitivity rather than true absence of effect.

Despite the limited statistical significance observed, the relationship between walking and cognition remains biologically plausible. Regular walking promotes cardiovascular fitness, enhances endothelial function, and improves cerebral perfusion, all of which are essential for sustaining neural metabolism in aging brains (Bolduc et al., 2013; Barnes and Corkery, 2018; Tari et al., 2025). Walking also modulates systemic inflammation and oxidative stress, reducing neurodegenerative risk (Cao et al., 2025). Moreover, it facilitates neurotrophic signaling pathways-particularly the upregulation of brain-derived neurotrophic factor (BDNF)-which supports synaptic plasticity and hippocampal neurogenesis (Shi et al., 2025). Taken together, these mechanisms support the existence of a walking–cognition relationship, even if such effects were not consistently detectable under the intervention conditions represented in the current evidence base. The absence of detectable cognitive improvement in this meta-analysis may therefore be attributable to methodological constraints, including short intervention duration, insufficient exercise intensity, and imprecise quantification of walking dose, rather than a lack of biological efficacy. Notably, walking speed or cadence-key determinants of both cardiovascular load and cognitive engagement—was inconsistently reported and could not be standardized across studies (Deshpande et al., 2009).

Another critical factor is the interaction between physical and cognitive engagement during walking. Dual-task walking paradigms, which require concurrent cognitive processing, have been associated with greater improvements in executive and attentional outcomes (Bensinger-Brody et al., 2025). However, only two included trials (Maki et al., 2012; Zukowski et al., 2022) directly assessed dual-task performance, and results were inconsistent, likely due to divergent task designs and outcome metrics. This limited and inconsistent evidence prevents firm conclusions regarding the added cognitive value of dual-task walking but highlights an important direction for future research. This highlights the need for standardized dual-task paradigms and suggests that integrating cognitive challenges into walking interventions may be essential for optimizing cognitive benefits. Moreover, virtual-reality treadmill interventions, by providing visuospatial feedback and task complexity, may offer superior cognitive stimulation, yet such approaches remain underrepresented in existing trials.

Several limitations should be considered when interpreting these findings. First, despite rigorous selection criteria, the number of eligible RCTs was small, and sample sizes within individual studies were limited, restricting the statistical power to detect small effect sizes and perform robust subgroup analyses. In particular, several cognitive domains were based on only two or three studies; therefore, the corresponding subgroup analyses and heterogeneity estimates should be regarded as exploratory rather than definitive. The considerable heterogeneity likely reflects variability in intervention design, participant characteristics, and assessment instruments, and control conditions. The included studies used substantially different comparators, including yoga, waiting-list controls, health education, normal walking, sitting, and conventional treadmill training. These control conditions differ markedly in their physiological and psychological activity levels; for example, yoga and conventional treadmill training may themselves exert cognitive or exercise-related effects, whereas sitting or waiting-list controls are largely inactive. Such variability may have diluted the estimated effects of walking training and contributed to the non-significant findings. For attention and executive function, dual-task performance, and drawing/executive tasks, heterogeneity was particularly high; therefore, due to excessive heterogeneity, the current evidence does not permit a reliable conclusion for these specific outcomes. Second, outcome indicators for cognitive function were highly integrated and diverse, including global screening tools and domain-specific tests, making it difficult to unify a single quantitative indicator across studies. This lack of standardization may have diluted domain-specific effects, thereby underestimating potential benefits of walking training. Third, although walking pace or step cadence is a critical determinant of exercise intensity and cognitive response, outcome indicators related to pace were inconsistently reported and could not be standardized across studies. Consequently, the influence of walking speed could not be adequately examined in this meta-analysis, which may have affected the precision of effect estimation. Fourth, multiple subgroup analyses were conducted without formal statistical correction for multiple testing, which may increase the risk of type I error; therefore, all subgroup findings should be interpreted cautiously. Given the limited number of studies within some domains, these findings were interpreted primarily in a descriptive and hypothesis-generating manner. Fifth, the inclusion of different walking modalities, such as overground walking, treadmill walking, interval walking, and virtual-reality-based walking training, as well as variations in supervision, further contributed to clinical and methodological heterogeneity. Lastly, the inclusion of different walking modalities (e.g., overground, treadmill, interval, and virtual-reality) and variations in supervision further contributed to heterogeneity. Lastly, publication bias remains a potential concern despite symmetrical funnel plots, as smaller negative trials may remain unpublished. Publication bias also remains a potential concern despite symmetrical funnel plots, as smaller negative trials may remain unpublished.

Future research should aim to address these limitations through large-scale, multicenter RCTs employing standardized protocols that quantify walking pace, intensity, and cognitive engagement. Consistent use of sensitive and domain-specific neuropsychological instruments is also essential to accurately assess cognitive change. Moreover, incorporating mechanistic biomarkers-such as neuroimaging, serum BDNF, and cerebral perfusion measures-could elucidate the physiological pathways linking walking to cognitive function.

## 5 Conclusion

This meta-analysis of nine RCTs involving 772 older adults found that walking-based interventions-including overground, interval, and virtual-reality treadmill training-did not significantly improve global or domain-specific cognitive outcomes compared with non-aerobic control conditions. Although slight trends toward enhanced attention, executive function, and global cognition were observed, these effects were not statistically significant and were accompanied by considerable heterogeneity. The findings suggest that walking alone showed no evidence of significantly improving cognitive function in older adults, and that programs incorporating higher intensity, longer duration, or cognitive engagement (such as dual-task or virtual-reality walking) should be further examined in future trials. However, limitations including small sample sizes, short intervention periods, inconsistent assessment tools, and heterogeneous outcome indicators-particularly the inability to unify walking pace or cadence measures-restrict the interpretability of results. Future studies should adopt standardized protocols quantifying walking intensity and cognitive engagement, employ sensitive domain-specific measures, and include mechanistic biomarkers to clarify the pathways linking walking exercise to cognitive function. Overall, walking remains a safe and accessible activity for older adults, but its independent role in cognitive improvement requires further high-quality evidence.

## Data Availability

The original contributions presented in the study are included in the article/Supplementary material, further inquiries can be directed to the corresponding author.
