# Supplementary material for: The impact of walking training on cognitive function in older adults: a meta-analysis
Source: Front Psychol. 2026 Jul 8;17:1720121. doi: 10.3389/fpsyg.2026.1720121 (PMC13388153; doi:10.3389/fpsyg.2026.1720121)
Supplement: Supplementary file 1 [file Table_1.DOCX]

Supplementary Material

| **Table S1** The searching strategy | | |
| --- | --- | --- |
| **Databases** | **Strategy** | **Literature number** |
| Pubmed | (((((Walking[MeSH Terms]) OR (Walking training[Title/Abstract])) OR (Walking[Title/Abstract])) OR (Ambulation[Title/Abstract])) AND ((((((((((Cognition[MeSH Terms]) OR (Cognition[Title/Abstract])) OR (Cognitions[Title/Abstract])) OR (Cognitive Function[Title/Abstract])) OR (Cognitive Functions[Title/Abstract])) OR (Function, Cognitive[Title/Abstract])) OR (Functions, Cognitive[Title/Abstract])) OR (Insight[Title/Abstract])) OR (Insights[Title/Abstract])) OR (Memory[Title/Abstract]))) AND (((Aged[MeSH Terms]) OR (Aged[Title/Abstract])) OR (Elderly[Title/Abstract])) | 4186 |
| Web of science | 1 "TS=(Walking training OR Walking OR Ambulation)  2 "TS=(Cognition OR Cognitions OR Cognitive Function OR Cognitive Functions OR Function, Cognitive OR Functions, Cognitive OR Insight OR Insights OR Memory)  3 "TS=(Aged OR Elderly)  4 "#3 AND #2 AND #1 | 6143 |
| Embase | 1 Walking training.ab,kf,ti. 1159  2 Walking.ab,kf,ti. 151032  3 Ambulation.ab,kf,ti. 25692  4 1 or 2 or 3 172199  5 Cognition.ab,kf,ti. 190267  6 Cognitions.ab,kf,ti. 10821  7 Cognitive Function.ab,kf,ti. 93996  8 Cognitive Functions.ab,kf,ti. 40610  9 Function, Cognitive.ab,kf,ti. 1353  10 Functions, Cognitive.ab,kf,ti. 298  11 Insight.ab,kf,ti. 388774  12 Insights.ab,kf,ti. 660302  13 Memory.ab,kf,ti. 473521  14 5 or 6 or 7 or 8 or 9 or 10 or 11 or 12 or 13 1690229  15 Aged.ab,kf,ti. 1198932  16 Elderly.ab,kf,ti. 475610  17 15 or 16 1574892  18 4 and 14 and 17 1999 | 1999 |
| Cochrane | #1 (Walking training):ti,ab,kw OR (Walking):ti,ab,kw OR (Ambulation):ti,ab,kw 38333  #2 (Cognition):ti,ab,kw OR (Cognitions):ti,ab,kw OR (Cognitive Function):ti,ab,kw OR (Cognitive Functions):ti,ab,kw OR (Function, Cognitive):ti,ab,kw 61564  #3 (Functions, Cognitive):ti,ab,kw OR (Insight):ti,ab,kw OR (Insights):ti,ab,kw OR (Memory):ti,ab,kw 57582  #4 #2 OR #3 97778  #5 (aged):ti,ab,kw OR (Elderly):ti,ab,kw 722321  #6 #1 AND #4 AND #5 1726 | 1726 |

| **Table S2** Walking dose parameters of included studies | | | | | | |
| --- | --- | --- | --- | --- | --- | --- |
| Study ID | Walking modality | Walking speed / cadence | Intensity | Weekly volume | Supervision status | Additional notes |
| Maki 2012 | Walking training | Not reported | Not reported | 90 min/week | Yes (small group) | Community-based |
| Oken 2006 | Walking exercise | Not reported | Target HR 70% max; Borg 6/7 | ≥300 min/week | Partially (weekly class) | Walking group of yoga trial |
| Okamoto 2019 | Interval walking training | Not reported | IWT: >70% / 40% VO₂peak | 180 min/week | No | Normal walking as control |
| Grede 2024 | Walking training | Not reported | Moderate (not specified) | Target 150 min/week | Yes (volunteer-supported) | POWER Study |
| Zheng 2024 | Cadence-controlled walking | 100 steps/min | Not reported | 30 min (single session) | Yes (laboratory) | Acute crossover study |
| Zukowski 2022 | VRTT | Self-paced | Not reported | 30 min (single session) | Yes (laboratory) | Control: conventional treadmill |
| Karmakar 2023 | Supervised group-based walking (SGBI) | Not reported | Not reported | 150–210 min/week (estimated) | Yes (professional trainer) | Control: non-supervised individual walking |
| Jamrasi 2024 | Walking + resistance (walking only) | Not reported | Moderate (RPE 12–14) | Not reported | Yes (main phase) | Combined exercise, walking-only group |
| **Note:** "Not reported" indicates that the parameter was not available in the original publication. "Estimated" values are calculated based on reported session frequency and duration. | | | | | | |

| 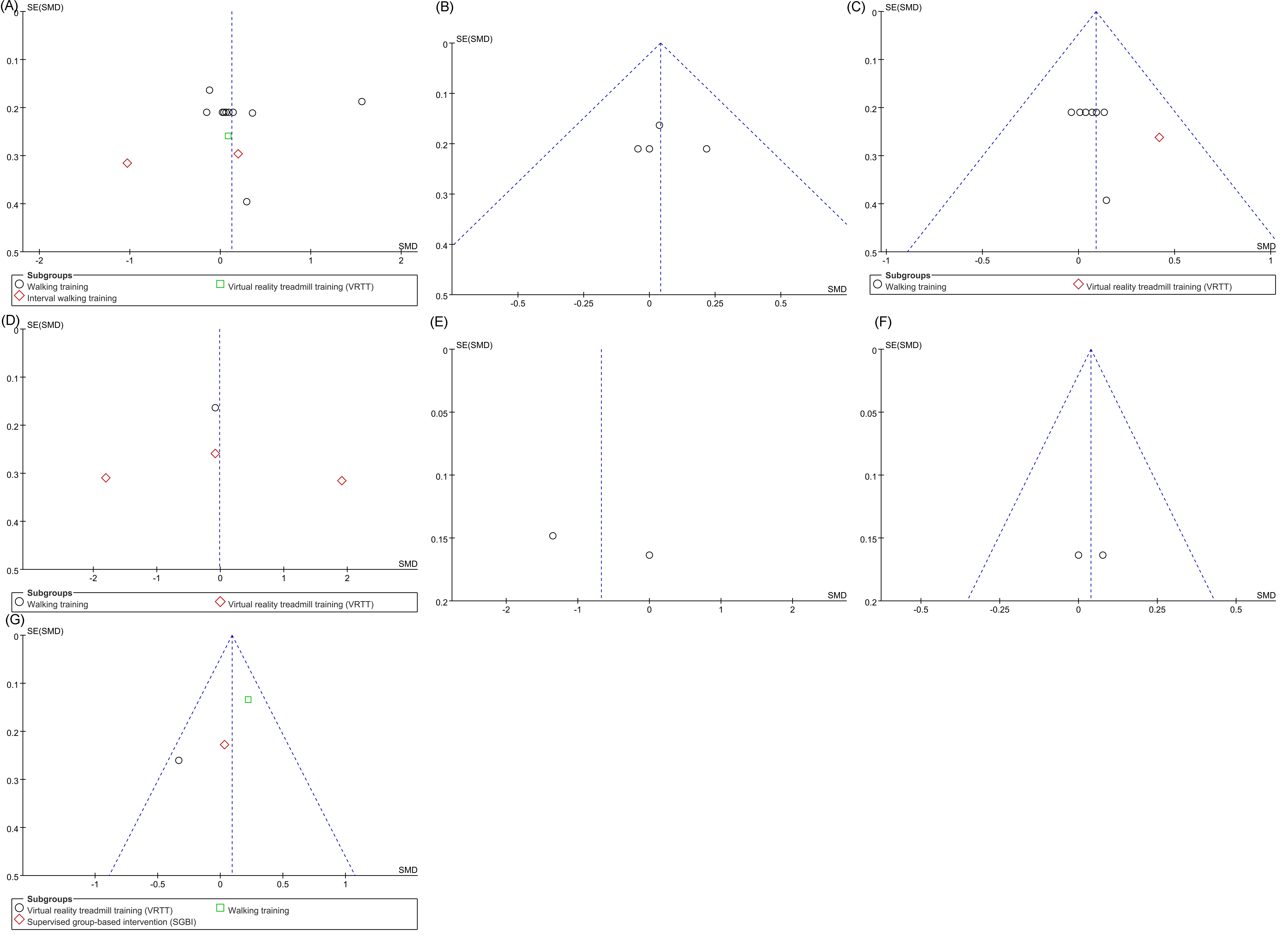 |
| --- |
| **Figure S1.** Funnel plot. (A). Attention and executive function; (B). Memory function; (C). Processing speed and reaction time; (D). Dual-task performance; (E). Drawing and executive tasks; (F). Substitution and symbol tasks; (G). Global cognitive screening outcomes. |

| 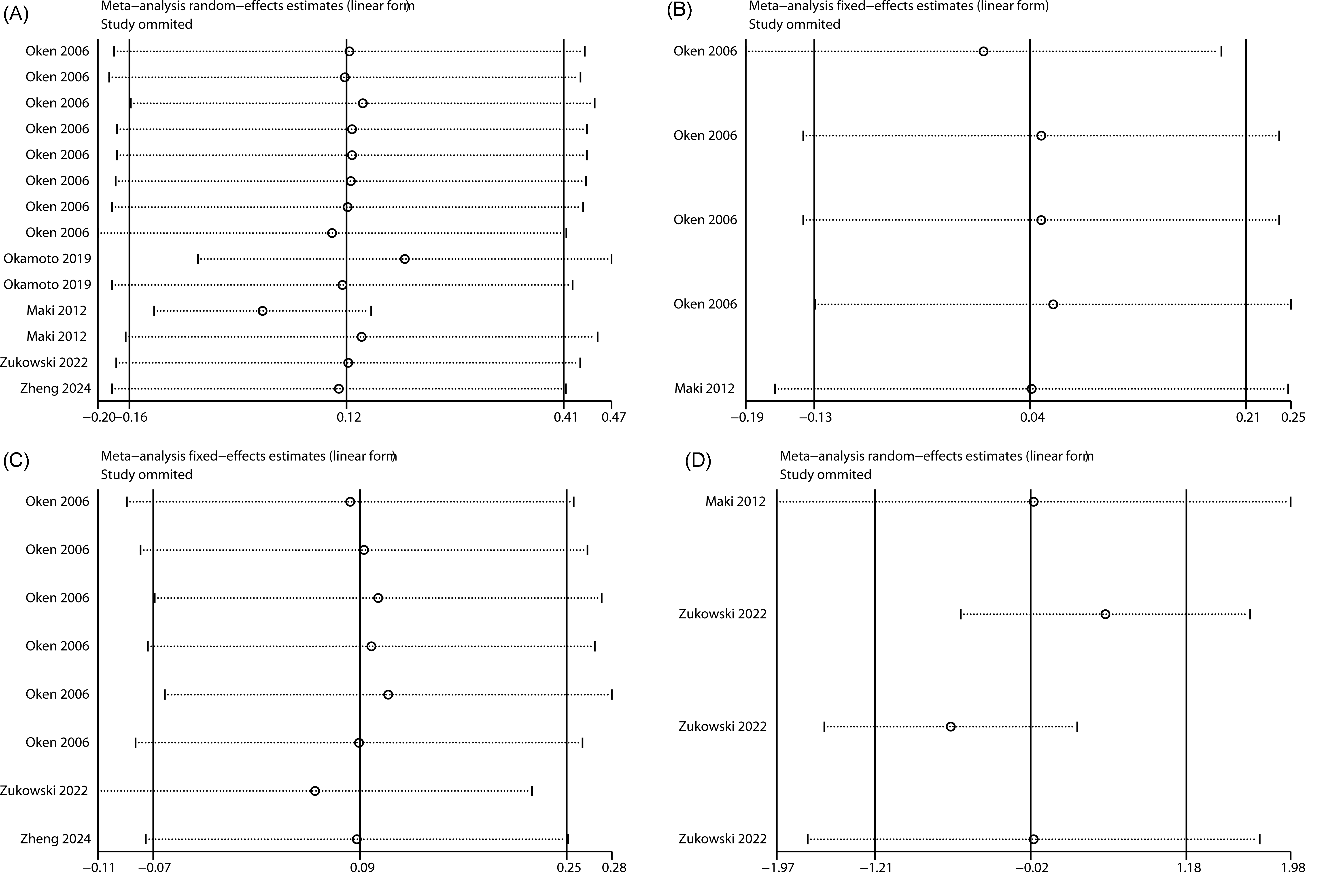 |
| --- |
| **Figure S2.** Sensitivity analysis. (A). Attention and executive function; (B). Memory function; (C). Processing speed and reaction time; (D). Dual-task performance. |
